# The association between red blood cell distribution width to albumin ratio and migraine: evidence from clinical and population-based cohorts

**DOI:** 10.3389/fneur.2026.1814482

**Published:** 2026-05-12

**Authors:** Rui Lu, Feng Li, Menghuan Yan, Xiaoxian Deng, Xintian Liu, Qi Chen, Haojie Zhang, Feng Zhu, Xuan Zheng, Gangcheng Zhang

**Affiliations:** 1 Structural Heart Disease Center, Zhongnan Hospital of Wuhan University, Wuhan, China; 2 Department of Neurology, Yangxin County People's Hospital, Huangshi, China

**Keywords:** body mass index, ICHD-3-diagnosed migraine, inflammation, National Health and Nutrition Examination Survey, severe headache, the red blood cell distribution width to albumin ratio

## Abstract

**Background:**

The red blood cell distribution width to albumin ratio (RAR) has been linked to inflammatory processes in neurological disorders, but its association with migraine-related outcomes remains unclear. This study aimed to explore the association between RAR and migraine.

**Methods:**

We investigated the association between RAR and ICHD-3-diagnosed migraine in a single-center retrospective observational cohort of consecutive adult patients presenting with headache to the Structural Heart Disease Center, Zhongnan Hospital of Wuhan University, between January 2024 and June 2025. Multivariable logistic regression analysis was employed to evaluate the association between RAR and migraine. P for trend across RAR quartiles was used to assess linear dose–response patterns, whereas restricted cubic splines (RCS) were used separately to examine potential nonlinearity. Subgroup analyses were performed to explore effect modification. For complementary epidemiological evidence, the relationship between RAR and self-reported severe headache or migraine was explored using a general population from the National Health and Nutrition Examination Survey (NHANES) database (1999–2004 cycles).

**Results:**

In the Chinese clinical cohort, higher RAR was associated with greater odds of ICHD-3-diagnosed migraine (odds ratio [OR] = 6.36, 95% confidence interval [CI]: 2.04–19.82, *p* = 0.001), with evidence of an overall association and no evidence of nonlinearity on RCS analysis (P overall = 0.004; P nonlinearity = 0.462). In the NHANES cohort, higher RAR was associated with greater odds of self-reported severe headache or migraine (OR = 1.23, 95% CI: 1.06–1.43, *p* = 0.006), also with no evidence of nonlinearity (P overall < 0.001; P nonlinearity = 0.902). A stronger association was observed among individuals aged <50 years in subgroup analyses (OR = 2.08, 95% CI: 1.74–2.48, *p* < 0.001). Exploratory analysis further suggested that BMI may partly account for the observed association in NHANES, possibly reflecting shared variance or potential confounding.

**Conclusion:**

Higher RAR was associated with migraine-related outcomes in the two complementary datasets. These findings are exploratory and hypothesis-generating and require confirmation in prospective studies.

## Introduction

Migraine is a highly prevalent neurovascular disorder associated with significant disability and quality-of-life impairment, affecting over 1 billion people globally. Clinically, it is characterized by moderate-to-severe throbbing headache, often accompanied by nausea, vomiting, photophobia, or phonophobia—symptoms that are typically exacerbated by routine physical activity (1). The 2025 Global Burden of Disease (GBD) study ranked headache disorders as the sixth most disabling group of conditions worldwide, underscoring its substantial socioeconomic burden (2). Epidemiological data further highlight its public health significance: the global prevalence of migraine ranges from 12 to 18%, with women facing a 2-to-3-fold higher risk than men, and migraine representing the primary cause of disability among individuals younger than 50 years (3–5).

Despite its high prevalence, migraine pathogenesis remains incompletely understood, involving diverse pathophysiological mechanisms and a complex etiological basis. Critically, a subset of migraine patients exhibits refractoriness to current therapeutic strategies, failing to achieve clinically meaningful headache improvement (6).

This challenge highlights the need to identify candidate markers associated with migraine-related outcomes, which may help improve understanding of disease heterogeneity. In recent years, inflammation has emerged as a key contributor to migraine pathogenesis. Preclinical studies have demonstrated that neuroinflammation in the intracranial meninges induces sensitization of trigeminal meningeal nociceptors, a pivotal event in migraine initiation and progression (7). Clinical evidence further supports this inflammatory link: migraine patients consistently show higher levels of high-sensitivity C-reactive protein (hs-CRP)—a well-validated systemic inflammatory marker—compared to headache-free controls, with elevated hs-CRP associated with increased migraine risk (8). Additionally, studies revealed associations between inflammation-related hematological parameters and migraine occurrence, collectively confirming the presence of a systemic inflammatory response in migraine patients (9, 10).

The red blood cell distribution width to albumin ratio (RAR), an integrative marker derived from routine laboratory tests, reflects both inflammatory status and nutritional condition—offering complementary insights that neither parameter alone can provide. Red blood cell distribution width (RDW), a measure of erythrocyte volume heterogeneity, has been linked to various systemic disorders and increased mortality risk (11). Serum albumin, conversely, serves as a crucial biomarker for evaluating inflammatory responses, as its synthesis is suppressed during acute or chronic inflammation. Together, RAR integrates these two dimensions to reflect multidimensional physiological dysfunction associated with inflammation and oxidative stress, making it a valuable tool for assessing immune status and immune responses (12). Recent studies have highlighted RAR as a candidate biomarker associated with adverse outcomes in multiple diseases, including stroke and cognitive impairment (13–16). However, the relationship between RAR and migraine—particularly whether RAR can serve as a reliable inflammatory-related biomarker for migraine—remains underexplored.

This study aimed to investigate the association between RAR and ICHD-3-diagnosed migraine in clinical headache patients, and to determine whether other clinical factors modify this relationship. Rather than serving as a direct validation analysis, the complementary cohort design addressed two distinct epidemiological questions: the Chinese clinical cohort examined the association of RAR with ICHD-3-diagnosed migraine among patients presenting with headache, whereas the NHANES cohort examined its association with self-reported severe headache or migraine in the general population. Together, these analyses provide complementary, hypothesis-generating evidence from two distinct settings.

## Methods

### Study design

This was a complementary cohort study including a Chinese clinical cohort and a NHANES general population cohort. The Chinese cohort was a single-center retrospective observational study conducted at the Structural Heart Disease Center (SHDC), Zhongnan Hospital of Wuhan University. We retrospectively screened consecutive adult patients who presented with headache as their primary complaint between January 2024 and June 2025. Patients admitted to the SHDC with headache as the primary complaint underwent a comprehensive clinical evaluation by a multidisciplinary team consisting of neurologists, psychologists, and cardiologists. The study protocol was approved by the Ethics Committee of Zhongnan Hospital of Wuhan University (approval no.: 2024295k). The informed consent was waived due to our retrospective study design. The inclusion criteria were as follows: (1) meeting the diagnostic criteria for migraine as specified in the International Classification of Headache Disorders, 3rd Edition (ICHD-3, 2018); (2) being 20 years of age or older. The exclusion criteria were: (1) headaches attributed to other etiologies; (2) missing values of RAR; (3) comorbid malignant tumors; (4) pregnancy; (5) incomplete clinical data. Complete clinical data were defined *a priori* as the availability of migraine outcome adjudication, RDW and serum albumin values required for RAR calculation, and all covariates included in the fully adjusted model (age, sex, BMI, smoking, alcohol intake, hypertension, diabetes, coronary heart disease, and stroke). Therefore, patients with missing RDW and/or albumin values, as well as those with missing covariates required for adjusted analyses, were excluded under a complete-case analysis strategy.

For complementary epidemiological analysis, data from the 1999–2004 cycles of the NHANES were retrieved, as questionnaires for assessing severe headache or migraine were available during this period. The detailed methodology and data collection protocols of NHANES have been approved by the National Center for Health Statistics (NCHS) Review Board and are publicly accessible via its official website. Informed consent was obtained from all participants, and no additional institutional review board (IRB) approval was required. For comprehensive methodological details regarding NHANES, please refer to the official website: https://www.cdc.gov/nchs/nhanes/index.htm. Participants were excluded if they were pregnant, younger than 20 years of age, or had missing exposure, outcome, or covariate data; thus, the NHANES analysis also used a complete-case approach.

### Assessment of headache outcomes

In the Chinese clinical cohort, the diagnosis of migraine was established through a comprehensive review of clinical records by two senior neurologists from the Multidisciplinary Team, strictly following the International Classification of Headache Disorders, 3rd Edition (ICHD-3, 2018) criteria. Key clinical features of were recorded, including the presence or absence of aura, episodic/chronic migraine classification (based on monthly attack frequency), and accompanying symptoms (nausea, photophobia, phonophobia) (17).

In the NHANES general population cohort, the outcome was defined as self-reported severe headache or migraine, based on an affirmative response to the questionnaire item: “Have you experienced a severe headache or migraine in the past three months?”. This single-item self-report measure is not a validated diagnostic tool for migraine. Accordingly, NHANES findings are interpreted conservatively as associations with self-reported severe headache or migraine rather than clinically confirmed migraine. Outcome misclassification may have biased the association toward or away from the null, and the diagnostic status of individual participants in the present NHANES dataset cannot be confirmed. We also cannot rule out the inclusion of other severe primary headache disorders.

Notably, the definition of control groups differed between the two cohorts. In the Chinese clinical cohort, controls were patients with headache who did not fulfill the ICHD-3 (2018) criteria for migraine. In the NHANES general population cohort, controls were individuals from the general population. This design allowed the association between RAR and headache-related outcomes to be examined in both a clinical headache population and the general population.

### Laboratory tests and RAR calculation

Laboratory tests results at admission, including blood routine, liver function, and renal function were collected for the Chinese cohort. The parameters used in the NHANES cohort were obtained from the laboratory data section of the NHANES database. The RAR was calculated as RDW (%) divided by serum albumin (g/dL).

### Covariates

In the Chinese cohort, covariates included gender, age, smoking status, alcohol drinking status, hypertension, diabetes, coronary heart disease, stroke, BMI and some laboratory measurements.

In the NHANES cohort, marital status (married, widowed, divorced, separated, never married, living with partner), race/ethnicity (non-Hispanic White, Mexican American, non-Hispanic Black, other Hispanic, and other races), education level (< high school, high school graduate, >high school), family income (PIR ≤ 1.3, 1.3–3.5, >3.5) and physical activity (inactive, moderate, vigorous) were included in addition to the covariates in the Chinese cohort. The classification of covariates was also consistent with the definition from prior studies (18).

### Statistical analysis

For the Chinese cohort, continuous variables were presented as mean ± standard deviation (SD) or median with interquartile range (IQR) as appropriate, while categorical variables were expressed as frequency (n) and percentage (%). Student’s t-tests and chi-square tests were used to compare group differences for continuous and categorical variables, respectively. For the NHANES cohort, all analyses conducted by incorporating appropriate sample weights. Data are presented as weighted mean ± standard error (SE) for continuous variables and as unweighted counts (*n*) with weighted percentages (%) for categorical variables.

The association between RAR and ICHD-3-diagnosed migraine (Chinese cohort) or self-reported severe headache or migraine (NHANES cohort) was examined using multivariable logistic regression. The events per variable (EPV) was >10 in all fully adjusted models to ensure model stability. The potential multicollinearity among the included covariates was evaluated using the variance inflation factor (VIF). All VIF values <5 indicated the absence of severe multicollinearity. In the Chinese cohort, Model 1 adjusted for no covariate; Model 2 adjusted for gender and age; Model 3 further included BMI, smoking, alcohol, hypertension, diabetes, coronary heart disease, and stroke. In NHANES, Model 1 was unadjusted; Model 2 adjusted for gender, age, and race; Model 3 additionally adjusted for education, marriage, PIR, BMI, smoking, alcohol, physical activity, hypertension, diabetes, coronary heart disease, and stroke. When RAR was treated as a continuous variable, the linear association for each one-unit increased in RAR and the risk of headache outcomes was analyzed. For categorical analysis, patients/participants were stratified into four quartiles, enabling comparisons of outcomes among groups with incrementally increased RAR exposure relative to a reference group. A *p*-value for trend across RAR quartiles was calculated to assess monotonic dose–response relationship. Restricted cubic splines (RCS) were then fitted separately to evaluate whether the association departed from linearity. Subgroup analyses and interaction tests were also conducted, and forest plots were generated to visualize the association across different subgroups.

Additionally, an exploratory associational intermediate analysis was performed in the NHANES cohort to examine whether BMI statistically explained part of the observed association between RAR and severe headache or migraine. This analysis is hypothesis-generating only and cannot support causal inference, because the cross-sectional design cannot establish the temporal ordering between variables. The proportion of the total observed association statistically explained by BMI was calculated. All statistical analyses were performed using Stata 17, R statistical software,^1^ and Empower Stats (http://www.empowerstats.com, X&Y Solutions, Inc., Boston, MA).

## Results

### Study population and baseline characteristics

**Figure 1:**
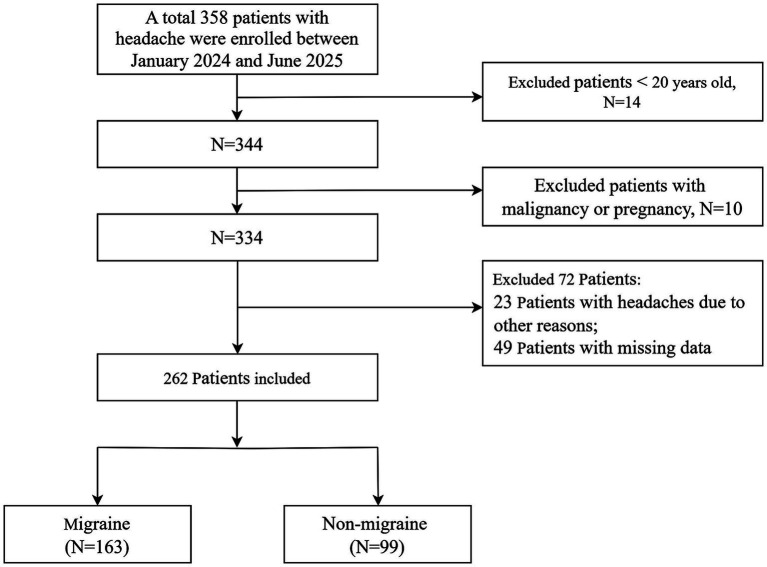
Flowchart of study population. Patients’ selection from Structural Heart Disease Center, Zhongnan Hospital of Wuhan University.

The retrospective observational study flow is illustrated in Figure 1. Initially, we reviewed clinical data of 358 consecutive patients who were admitted with headache as the primary complaint. Among these individuals, 14 were excluded due to being younger than 20 years, 10 were excluded for comorbid malignancy or pregnancy, and 23 were excluded after confirming their headaches caused by new-onset ischemic stroke (*n* = 7), neurovascular disease (*n* = 9), and psychiatric disorders (*n* = 7). Additionally, 49 patients were excluded under the complete-case strategy because of incomplete clinical data, defined as missing RDW and/or albumin values required for RAR calculation, missing migraine outcome adjudication, or missing covariates required for the adjusted analyses. After applying the above exclusion criteria, the Chinese cohort was finally composed of 262 patients, of whom 163 (62.2%) met the ICHD-3 (2018) diagnostic criteria for migraine. The clinical features of migraine patients in the Chinese cohort were as follows: 15 (9.2%) cases with aura; 22 (13.5%) cases of episodic migraine (monthly attacks < 15 days), 98 (60.1%) cases of chronic migraine (meeting ICHD-3 criteria: monthly headache days ≥ 15 for ≥ 3 months, with ≥ 8 days of migraine-feature headache); 12 (7.4%) patients with accompanying nausea/vomiting, and 16 (9.8%) patients with photophobia/phonophobia. As noted, the low prevalence of accompanying symptoms likely reflects underreporting in retrospective routine clinical documentation. Baseline characteristics of the cohort are summarized in Table 1. Hypertension (*n* = 44, 16.79%) and stroke (*n* = 60, 22.9%) were the most prevalent comorbidities in the entire cohort. Notably, non-migraine patients had a higher prevalence of hypertension (24.24% vs. 12.27%, *p* = 0.012), diabetes (11.11% vs. 3.07%, *p* = 0.008), and stroke (39.39% vs. 12.88%, *p* < 0.001) when compared with migraine patients. In contrast, migraine patients exhibited a significant younger age (44.90 ± 12.39 vs. 50.98 ± 11.79, *p* < 0.001) and a higher female proportion (79.75% vs. 46.46%, *p* < 0.001) than non-migraine patients. In laboratory measurements, migraine patients showed significantly elevated levels of total cholesterol (4.33 ± 0.85 mmol/L vs. 4.06 ± 0.95 mmol/L, *p* = 0.019), high density lipoprotein (1.29 ± 0.33 mmol/L vs. 1.21 ± 0.36 mmol/L, *p* = 0.049), low density lipoprotein (2.64 ± 0.71 mmol/L vs. 2.41 ± 0.78 mmol/L, *p* = 0.018), red blood cell [(4.29 ± 0.42) × 10^12^/L vs. (4.40 ± 0.41) × 10^12^/L, *p* = 0.024] and RAR (3.07 ± 0.37 vs. 2.97 ± 0.28, *p* = 0.030) compared with non-migraine patients.

**Table 1:** Baseline characteristics of the Chinese cohort.

Characteristic	Total	Migraine	Non-migraine	*p* value
	(*n* = 262)	(*n* = 163)	(*n* = 99)	
General				
Age (years)	47.20 ± 12.50	44.90 ± 12.39	50.98 ± 11.79	<0.001
BMI (Kg/m^2^)	23.71 ± 3.60	23.74 ± 3.71	23.64 ± 3.44	0.830
Gender (*n*, %)				<0.001
Male	86 (32.82)	33 (20.25)	53 (53.54)	
Female	176 (67.18)	130 (79.75)	46 (46.46)	
Personal history				
Alcohol intake (*n*, %)				0.133
No	250 (95.42)	158 (96.93)	92 (92.93)	
Yes	12 (4.58)	5 (3.07)	7 (7.07)	
Smoking (*n*, %)				0.071
No	251 (95.8)	159 (97.55)	92 (92.93)	
Yes	11 (4.2)	4 (2.45)	7 (7.07)	
Hypertension (*n*, %)				0.012
No	218 (83.21)	143 (87.73)	75 (75.76)	
Yes	44 (16.79)	20 (12.27)	24 (24.24)	
Diabetes (*n*, %)				0.008
No	246 (93.89)	158 (96.93)	88 (88.89)	
Yes	16 (6.11)	5 (3.07)	11 (11.11)	
Coronary heart disease (*n*, %)				0.779
No	255 (97.33)	159 (97.55)	96 (96.97)	
Yes	7 (2.67)	4 (2.45)	3 (3.03)	
Stroke (*n*, %)				<0.001
No	202 (77.1)	142 (87.12)	60 (60.61)	
Yes	60 (22.9)	21 (12.88)	39 (39.39)	
Laboratory measurements				
WBC (10^9^/L)	6.02 ± 1.68	5.91 ± 1.67	6.21 ± 1.68	0.174
RBC (10^12^/L)	4.33 ± 0.42	4.29 ± 0.42	4.40 ± 0.41	0.024
HGB (g/L)	132.07 ± 13.41	129.71 ± 12.82	135.94 ± 13.50	<0.001
PLT (10^9^/L)	229.08 ± 58.39	228.15 ± 60.15	230.63 ± 55.64	0.740
NEUT (10^9^/L)	3.70 ± 1.33	3.62 ± 1.34	3.84 ± 1.32	0.188
LYM (10^9^/L)	1.76 ± 0.60	1.76 ± 0.58	1.76 ± 0.63	0.953
RDW (%)	13.25 ± 1.11	13.31 ± 1.27	13.15 ± 0.77	0.257
TC (mmol/L)	4.23 ± 0.90	4.33 ± 0.85	4.06 ± 0.95	0.019
TG (mmol/L)	1.59 ± 1.33	1.47 ± 1.04	1.77 ± 1.69	0.078
HDL (mmol/L)	1.26 ± 0.35	1.29 ± 0.33	1.21 ± 0.36	0.049
LDL (mmol/L)	2.55 ± 0.74	2.64 ± 0.71	2.41 ± 0.78	0.018
AST (U/L)	22.22 ± 11.33	22.04 ± 11.68	22.52 ± 10.78	0.739
Creatinine (umol/L)	66.31 ± 48.99	65.68 ± 61.23	67.34 ± 13.91	0.792
Albumin (g/dL)	4.42 ± 0.31	4.40 ± 0.29	4.45 ± 0.34	0.233
RAR	3.03 ± 0.34	3.07 ± 0.37	2.97 ± 0.28	0.030

AST, aspartate transaminase; BMI, Body Mass Index; RDW, red cell distribution width; RAR, red blood cell distribution width to albumin ratio; HGB, hemoglobin; HDL, high density lipoprotein; LDL, low density lipoprotein; LYM, lymphocyte; NEUT, neutrophil; PLT, Platelets; RBC, red blood cell; WBC, white blood cell; TC, total cholesterol; TG, triglyceride.

For the NHANES cohort, 11,898 participants were included after applying the exclusion criteria (Supplementary Figure S1), among whom 2,385 (20.0%) reported self-reported severe headache or migraine. The lifestyle and comorbidity profile of the NHANES cohort differed from that of the Chinese clinical cohort: the most prevalent lifestyle factor was alcohol intake (*n* = 7,735, 65.01%), and the prevalence of smoking (*n* = 8,627, 49.75%) was also higher in the NHANES cohort. Nevertheless, participants with the severe headache or migraine in the NHANES cohort exhibited a similar trend to those with ICHD-3-diagnosed migraine in the Chinese cohort, including younger age, female predominance, and significantly higher RAR levels (Supplementary Table S1). Additionally, participants with severe headache or migraine had a significantly higher BMI than participants without this outcome (28.9 ± 7.0 vs. 27.9 ± 6.0, *p* < 0.001).

### Association between RAR and headache outcomes

**Figure 2:**
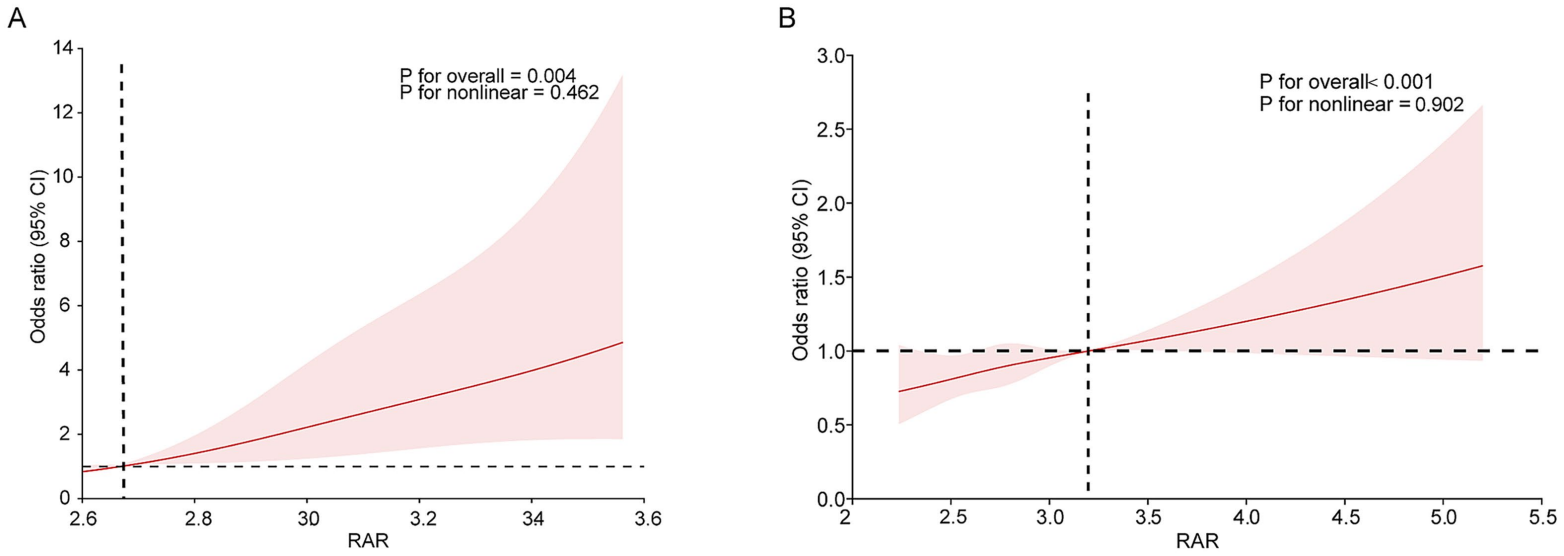
Restricted cubic spline curve (RCS) plot of the association between RAR and the odds of ICHD-3-diagnosed migraine in the Chinese cohort **(A)** and self-reported severe headache or migraine in the NHANES cohort **(B)**. The red area represents the 95% confidence interval; the red lines represent inflection points.

In the Chinese cohort, multivariable logistic regression analyses revealed a significant positive association between continuous RAR and ICHD-3-diagnosed migraine across all models (Table 2). EPV analysis confirmed that the number of migraine cases per covariate was 163, 81.5, and 18.1 across Model 1 to 3, indicating adequate sample size and no model overfitting; VIF values ranged from 1.14 to 1.21, arguing against multicollinearity. After adjusted for gender and age in Model 2, higher RAR was associated with greater odds of migraine (OR = 4.34, 95%CI: 1.53–12.33, *p* = 0.006). The association remained significant after full adjustment (OR = 6.36, 95%CI:2.04–19.82, *p* = 0.001). The linear trend was significant in Model 2 (OR = 1.39, 95%CI: 1.07–1.80, *p* = 0.014) and Model 3 (OR = 1.49, 95%CI:1.13–1.97, *p* = 0.004). RCS analysis showed evidence of an overall association without evidence of nonlinearity (P for overall = 0.004, P for non-linearity = 0.462, Figure 2A). However, categorical analysis by quartiles demonstrated that the migraine risk peaked at the third quartile (Q3) and attenuated in the highest quartile (Q4) across all models, suggesting a potential plateau effect at extreme RAR levels.

**Table 2:** The association between RAR and ICHD-3-diagnosed migraine in Chinese cohort.

Characteristic	Model 1		Model 2		Model 3	
	OR (95%CI)	*p* value	OR (95%CI)	*p* value	OR (95%CI)	*p* value
RAR	2.57 (1.08–6.12)	0.033	4.34 (1.53–12.33)	0.006	6.36 (2.04–19.82)	0.001
Categories						
Q1	1 (Ref)		1(Ref)		1(Ref)	
Q2	1.54 (0.77–3.09)	0.220	1.68 (0.78–3.63)	0.184	1.60 (0.71–3.59)	0.254
Q3	2.41 (1.16–4.98)	0.018	3.45 (1.51–7.86)	0.003	4.26 (1.77–10.25)	0.001
Q4	1.65 (0.82–3.30)	0.160	2.36 (1.06–5.26)	0.036	2.79 (1.19–6.52)	0.018
Trend test	1.22 (0.97–1.52)	0.087	1.39 (1.07–1.80)	0.014	1.49 (1.13–1.97)	0.004

Model 1 (Unadjusted model): No covariate adjustment. Model 2 (Partially adjusted model): Adjusted for gender, age. Model 3 (Fully adjusted model): Adjusted for gender, age, BMI, smoking, alcohol, hypertension, diabetes, coronary heart disease, stroke. RAR Q1 = 2.83, Q2 = 3.00, Q3 = 3.18, Q4 = 5.43. BMI, body mass index; CI: confidence interval; OR: odds ratio; Q: quartiles; RAR: ratio of red blood cell distribution width to albumin; Ref: reference.

Similar results were observed in the NHANES cohort. Continuous analysis showed greater odds of self-reported severe headache or migraine with higher RAR across all models (Table 3). The RCS plot showed evidence of an overall association but no departure from linearity (P for overall <0.001, P for non-linearity = 0.902, Figure 2B). In categorical analysis, while the middle quartiles (Q2 and Q3) showed fluctuating or plateauing risk levels, the highest RAR quartile (Q4) consistently exhibited the most pronounced risk of severe headache or migraine across all models (Table 3). In addition, BMI ≥ 30 remained independently associated with greater odds of self-reported severe headache or migraine across progressively adjusted models, with P for trend < 0.001 in all three models. These findings indicate that BMI is an important covariate in the observed RAR-headache association, but they should not be interpreted as evidence of a mechanistic pathway.

**Table 3:** The association between RAR and self-reported severe headache or migraine in the NHANES cohort.

Characteristic	Model 1		Model 2		Model 3	
	OR (95%CI)	*p* value	OR (95%CI)	*p* value	OR (95%CI)	*p* value
RAR	1.42 (1.26–1.60)	<0.001	1.5 (1.31–1.73)	<0.001	1.23 (1.06–1.43)	0.006
Categories						
Q1	1 (Ref)		1 (Ref)		1 (Ref)	
Q2	1.21 (1.04–1.41)	0.014	1.27 (1.08–1.49)	0.004	1.17 (0.99–1.38)	0.065
Q3	1.20 (1.03–1.40)	0.021	1.34 (1.13–1.58)	0.001	1.13 (0.95–1.35)	0.160
Q4	1.55 (1.33–1.81)	<0.001	1.68 (1.40–2.00)	<0.001	1.31 (1.09–1.59)	0.005
Trend test	1.14 (1.08–1.20)	<0.001	1.17 (1.11–1.24)	<0.001	1.08 (1.02–1.15)	0.011
BMI^a^	1.02 (1.01–1.03)	<0.001	1.03 (1.02–1.03)	<0.001	1.02 (1.01–1.03)	<0.001
Categories						
<25	1 (Ref)		1 (Ref)		1 (Ref)	
25–30	0.96 (0.83–1.10)	0.526	1.18 (1.02–1.36)	0.026	1.14 (0.99–1.32)	0.071
≥30	1.32 (1.15–1.51)	<0.001	1.48 (1.28–1.70)	<0.001	1.33 (1.15–1.54)	<0.001
Trend test	1.03 (1.01–1.04)	<0.001	1.04 (1.02–1.05)	<0.001	1.03 (1.01–1.04)	<0.001

Model 1 (Unadjusted model): No covariate adjustment. Model 2 (Partially adjusted model): Adjusted for gender, age, and race. Model 3 (Fully adjusted model): Adjusted for gender, age, race, education, marriage, poverty-income ratio, BMI, smoking, alcohol, physical activity, hypertension, diabetes, coronary heart disease, stroke. RAR Q1 = 2.73, Q2 = 2.93, Q3 = 3.15, Q4 = 8.37. BMI, body mass index; CI: confidence interval; OR: odds ratio; Q: quartiles; RAR, ratio of red blood cell distribution width to albumin; Ref: reference.

a Excluded when BMI was analyzed as the exposure.

### Subgroup and interaction analysis

**Figure 3:**
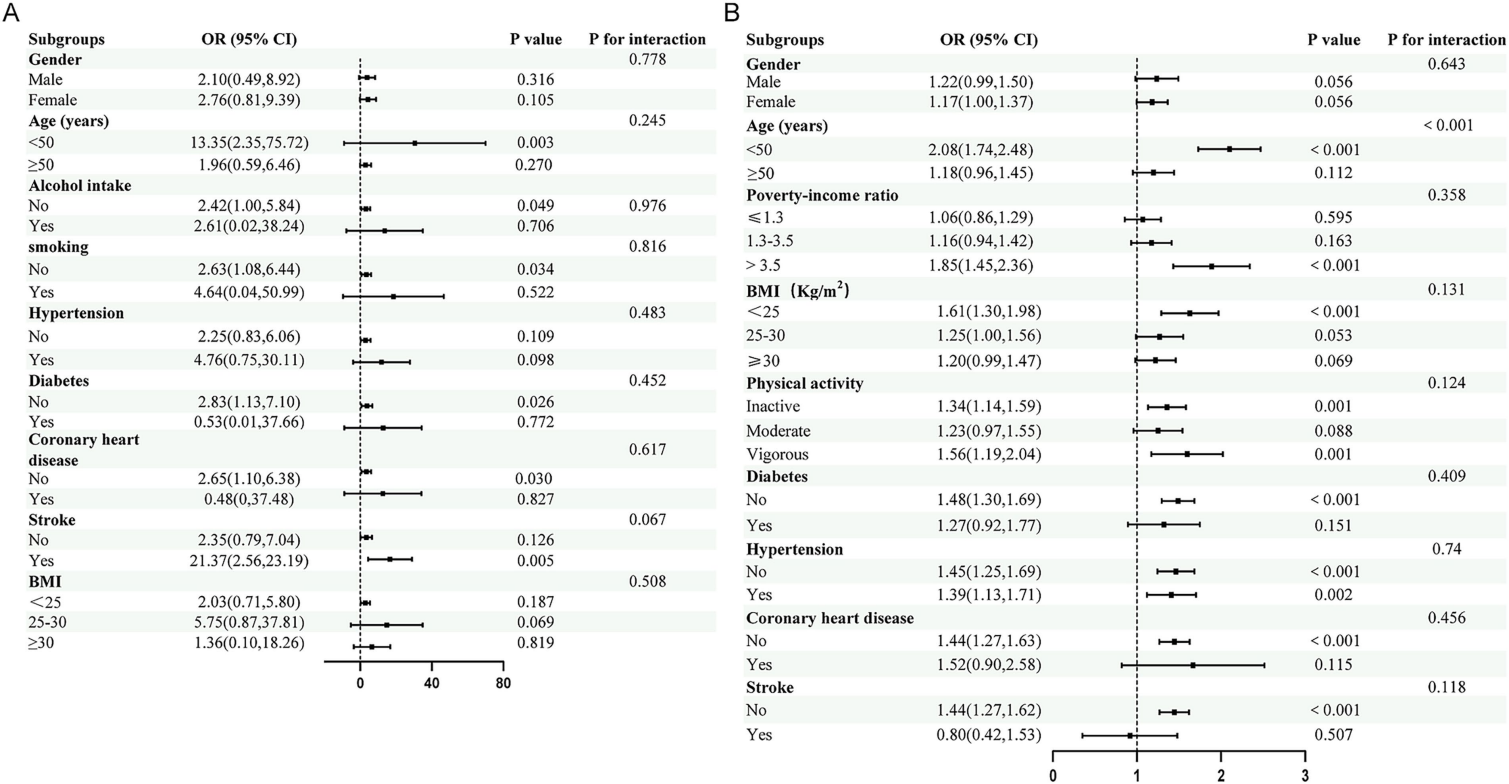
Forest plots of Subgroup and interaction analysis for the association between RAR and the odds of ICHD-3-diagnosed migraine in the Chinese cohort **(A)** and self-reported severe headache or migraine in the NHANES cohort **(B)**. Subgroup findings should be interpreted cautiously, particularly in the Chinese cohort where several estimates were imprecise.

In the Chinese cohort, some subgroup estimates, including those for age <50 years (OR = 13.35, 95%CI:2.35–75.72, *p* = 0.003) and history of stroke (OR = 21.37, 95%CI:2.56–23.19, *p* = 0.005), were large but imprecise and should be interpreted cautiously as exploratory findings (Figure 3A). In the NHANES cohort, a positive association between RAR and self-reported severe headache or migraine was observed in participants with PIR > 3.5 (OR = 1.85, 95%CI:1.45–2.36, *p* < 0.001), BMI < 25 (OR = 1.61, 95%CI:1.30–1.98, *p* < 0.001), and age<50 years (OR = 2.08, 95%CI:1.74–2.48, *p* < 0.001, Figure 3B). In contrast, history of stroke was not associated with higher odds of self-reported severe headache or migraine in NHANES (OR = 0.80, 95%CI:0.42–1.53, *p* = 0.507). A significant interaction between age and RAR was detected in NHANES (*p* < 0.01).

### Exploratory associational analysis in the NHANES cohort

**Figure 4:**
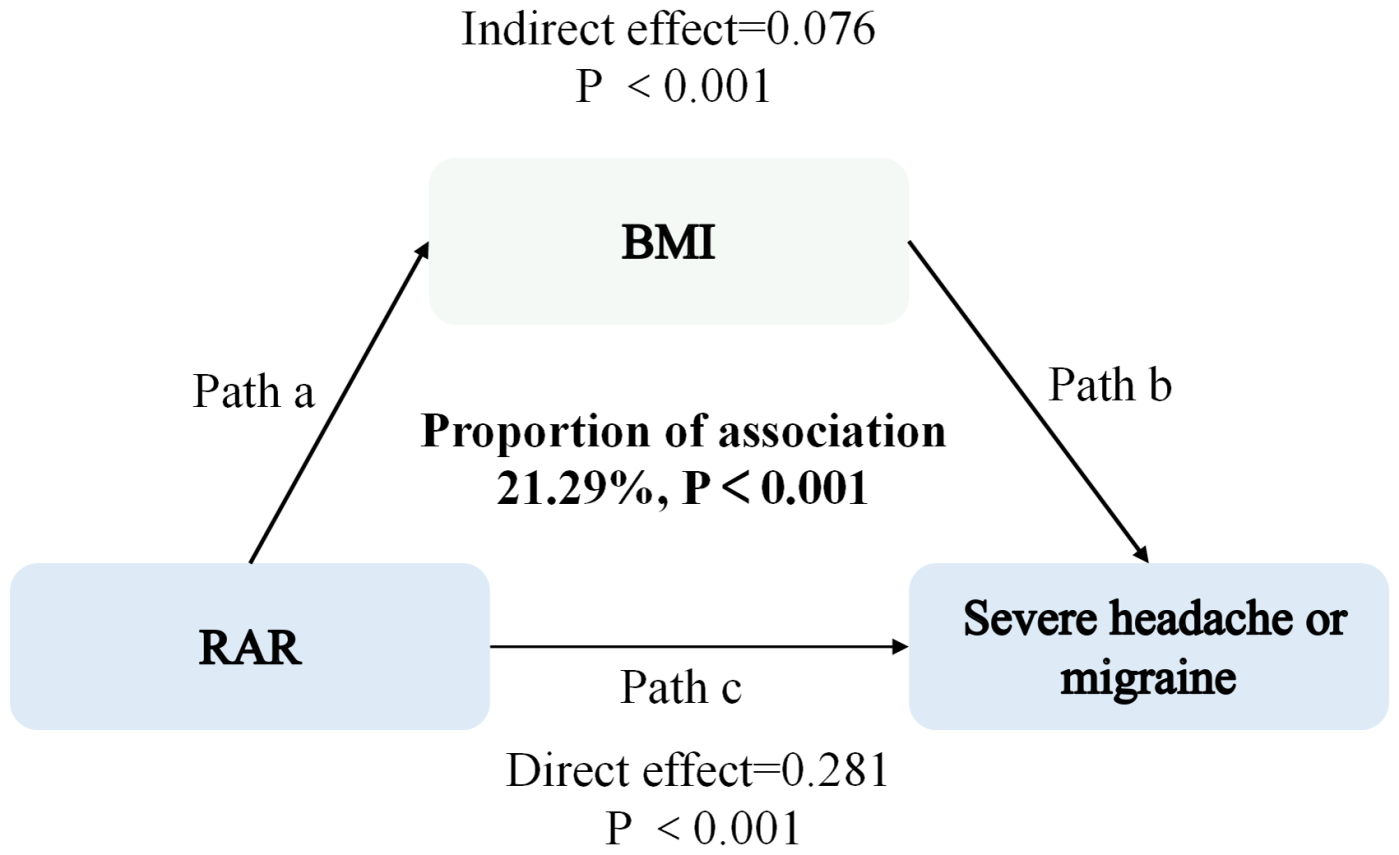
Exploratory associational model in the NHANES cohort examining RAR, BMI, and self-reported severe headache or migraine. RAR was specified as the independent variable, self-reported severe headache or migraine as the dependent variable, and BMI as a variable included in the exploratory model. Path a represents the association between RAR and BMI; path b represents the association between BMI and self-reported severe headache or migraine; and path c represents the direct association of RAR with self-reported severe headache or migraine. This model is exploratory and reflects associational relationships only; it cannot support inference about causality, mediation, or mechanism.

We performed an exploratory associational analysis to examine whether BMI statistically explained part of the observed association between RAR and the NHANES self-reported severe headache or migraine outcome. This analysis was purely hypothesis-generating and cannot infer causal effects, as the cross-sectional design cannot establish the temporal ordering between variables required for causal inference. In the NHANES cohort, attenuation after incorporating BMI may reflect shared variance, overlap in risk profiles, or potential confounding rather than a confirmed intermediate pathway. In this exploratory model, BMI accounted for 21.29% of the total observed association between RAR and self-reported severe headache or migraine (Figure 4).

## Discussion

Migraine affects more than one billion people worldwide, yet clinically validated markers associated with migraine-related outcomes remain limited. To our knowledge, the present study is the first to report a significant positive association between RAR and migraine-related outcomes. In this retrospective clinical cohort, higher RAR was associated with greater odds of ICHD-3-diagnosed migraine, and a directionally similar association was observed for self-reported severe headache or migraine in NHANES. Subgroup analyses suggested that the association may be more apparent among individuals aged <50 years, although these findings, particularly in the Chinese cohort, should be interpreted cautiously. Exploratory analysis in the NHANES cohort further showed that adjustment for BMI explained part of the observed RAR-outcome association, but this finding should be interpreted cautiously.

Migraine is characterized by recurrent severe headaches, however, the underlying mechanisms remain incompletely understood. Previous studies suggest that RDW and albumin may reflect inflammatory and oxidative stress states that could be relevant to migraine-related biology (19–21). Elevated RDW has been linked to increased release of pro-inflammatory cytokines and reactive oxygen species (ROS), which could hypothetically contribute to trigeminovascular activation (22). Low albumin levels may reduce antioxidant capacity and may also be relevant to oxidative stress-related process (23, 24). However, the present observational data do not permit mechanistic inference, and these considerations should be viewed as hypothesis-generating only.

In the present study, elevated RAR was positively associated with headache outcomes in two demographically distinct cohorts. These findings are consistent with the possibility that RAR captures biological information related to inflammatory and nutritional status, but they do not establish that RDW and albumin act through a specific mechanistic pathway in migraine. Thus, RAR should presently be interpreted as an integrated observational biomarker rather than as evidence of causality. Further *in vivo* and *in vitro* experiments are warranted to evaluate the biological mechanisms that may underlie this association.

For patients with migraine comorbid with patent foramen oval (PFO) (25), cardiovascular specialists, particularly those in structural heart disease centers, focus on screening for migraine-related risk factors and conducting etiological assessments. In the present study, the Chinese clinical cohort was recruited from a specialized structural heart disease center, where migraine diagnoses were established through multidisciplinary evaluation. This recruitment setting may limit the generalizability to migraine population managed in neurology clinics. The two cohorts also used different control groups to address distinct epidemiological questions, which likely contributed to differences in the magnitude of the observed odds ratios. The Chinese clinical cohort compared migraine patients with non-migraine headache patients, thereby reducing confounding related to headache versus non-headache status; however, because of substantial baseline imbalances, residual confounding in this cohort cannot be excluded despite multivariable adjustment. By contrast, the NHANES analysis compared individuals with self-reported severe headache or migraine with the general population. For conservative interpretation, the NHANES findings should be generalized to self-reported severe headache or migraine rather than clinically confirmed migraine. Despite these differences, directionally similar associations were observed across the two datasets. These findings provide complementary evidence for a possible relationship between RAR and headache-related outcomes, but they should not be interpreted as direct validation or immediate clinical applicability.

On a global scale, migraine persists as the sixth leading cause of disability across all age groups and ranks first among women under 50 years of age (26, 27). A household-based questionnaire survey encompassing 15,000 U.S. households demonstrated that the prevalence of migraine in women is two to threefold higher than in men, with the peak prevalence observed among individuals aged 35–45 years (28). Specifically, in our cohort, the migraine group had a higher proportion of females (79.75% vs. 46.46% in the non-migraine group), a younger age (44.90 ± 12.39 years vs. 50.98 ± 11.79 years), a lower prevalence of hypertension (12.27% vs. 24.24%), and a lower prevalence of stroke (12.88% vs. 39.39%) compared with the non-migraine group. In contrast, no significant interaction between RAR and demographic or clinical factors was detected in the Chinese clinical cohort, a finding that may be attributable to the relatively small sample size of this study population. By contrast, interaction analyses performed on the NHANES cohort revealed that the positive association between RAR and self-reported severe headache or migraine is notably more pronounced in participants under 50 years of age. Collectively, these results align with previously reported evidence on the relationship between age and migraine, and suggest that the association may be more apparent among individuals aged <50 years, although these subgroup findings require cautious interpretation, particularly in the smaller Chinese cohort.

Exploratory analysis in the NHANES cohort suggested that BMI accounted for approximately 21.29% of the observed association between RAR and self-reported severe headache or migraine. Given the cross-sectional design, this attenuation after BMI adjustment is more appropriately interpreted as reflecting shared variance, overlap in inflammatory-metabolic profiles, or potential confounding rather than evidence that BMI lies on a confirmed mechanistic pathway. Previous studies suggest that elevated RAR reflects systemic inflammation and nutritional impairment, which can lead to abnormal lipid metabolism and weight gain (29–31); meanwhile, obese individuals have increased adipose tissue-derived pro-inflammatory cytokines (e.g., TNF-*α*, IL-6) (32–34), which further exacerbate systemic inflammation and neurogenic inflammation cycle. Nonetheless, the present data cannot determine the directionality or causal structure linking RAR, BMI, and headache outcomes.

However, it is critical to note that the exploratory associational analysis in the present study is purely associational and cannot infer causal effects, as the NHANES cohort is a cross-sectional design where RAR, BMI, and self-reported severe headache or migraine were measured simultaneously. Therefore, the observed indirect statistical effect should not be interpreted as mediation. Instead, it indicates that BMI is an important covariate in the RAR-headache association and warrants further evaluation in prospective studies with clear temporal ordering and repeated measurements.

### Strengths and limitations

A key strength of the present study is its complementary cohort design: the Chinese clinical cohort identified the association between RAR and ICHD-3-diagnosed migraine in patients presenting with headache, while the NHANES cohort provided complementary epidemiological evidence regarding self-reported severe headache or migraine in the general population. In addition, we performed EPV and VIF assessments to support model stability and used RCS analysis to explore potential nonlinearity. We also collected detailed clinical features of migraine patients in the Chinese cohort, which adds clinical context to the findings.

Nonetheless, several critical limitations of this study must be addressed. First, the Chinese clinical cohort was recruited from a single structural heart disease center, which may introduce selection bias and limit the generalizability of our findings to broader migraine populations. In addition, this cohort was retrospective and analyzed using a complete-case approach, so exclusion of patients with missing RAR components or covariate data may also have introduced selection bias. Second, the NHANES outcome was defined by a single self-reported question of “severe headache or migraine in the past three months.” For conservative interpretation, this endpoint should be regarded as self-reported severe headache or migraine rather than clinically confirmed migraine. Outcome misclassification is possible, and we cannot rule out the inclusion of other severe primary headache disorders. Third, the retrospective observational design of the Chinese cohort and the cross-sectional design of the NHANES cohort preclude causal inference and may have led to underreporting of photophobia, phonophobia, and nausea in routine documentation. Moreover, the exploratory analysis involving BMI cannot establish mediation or mechanism because temporal ordering is unavailable. Finally, the relatively limited sample size and substantial baseline imbalances in the Chinese cohort may have contributed to imprecision at the extreme end of the RAR distribution. Although multivariable regression was used to adjust for measured covariates, residual confounding cannot be fully excluded.

Accordingly, future investigations should adopt well-defined, large-scale, multicenter, prospective study designs to confirm the observed associations and to improve understanding of the biological processes that may underlie them. Additionally, studies using propensity score matching (PSM) or inverse probability weighting (IPW) to reduce baseline imbalances, and Mendelian randomization to explore potential causal relationships are warranted. Further research is also needed to determine whether RAR has reproducible associations across broader populations and whether its longitudinal behavior is informative in future prospective studies.

## Conclusion

In summary, this complementary cohort study showed a positive association between RAR and ICHD-3-diagnosed migraine in a clinical headache cohort and between RAR and self-reported severe headache or migraine in the general population. Subgroup analyses suggested possible heterogeneity by age, but these findings should be interpreted cautiously. Exploratory analysis suggests that BMI is an important covariate in this association, but its role should not be interpreted as evidence of a confirmed mechanistic pathway. Future prospective studies are needed to confirm these associations and to further investigate the biological processes that may underlie them.

## Data Availability

The original contributions presented in the study are included in the article/Supplementary material, further inquiries can be directed to the corresponding authors.

## Footnotes

1
https://www.r-project.org/

## Glossary

RAR: red blood cell distribution width to albumin ratio
BMI: body mass index
NHANES: National Health and Nutrition Examination Survey
RCS: restricted cubic spline
OR: odds ratio
GBD: Global Burden of Disease
hs-CRP: high-sensitivity C-reactive protein
RDW: red blood cell distribution width
ICHD-3 International Classification of Headache Disorders, 3rd Edition
NCHS: National Center for Health Statistics
AMPP: American Migraine Prevalence and Prevention
IRB: institutional review board
PIR: poverty income ratio
SD: standard deviation
IQR: interquartile range
CI: confidence intervals
SE: standard error
EPV: events per variable
VIF: variance inflation factor
ROS: reactive oxygen species
TNF-*α*: tumor necrosis factor-α
IL-6: interleukin-6
PSM: propensity score matching
IPW: inverse probability weighting
