# Supplementary figures and images for: The association between red blood cell distribution width to albumin ratio and migraine: evidence from clinical and population-based cohorts
Source: Front Neurol. 2026 May 12;17:1814482. doi: 10.3389/fneur.2026.1814482 (PMC13201144; doi:10.3389/fneur.2026.1814482)

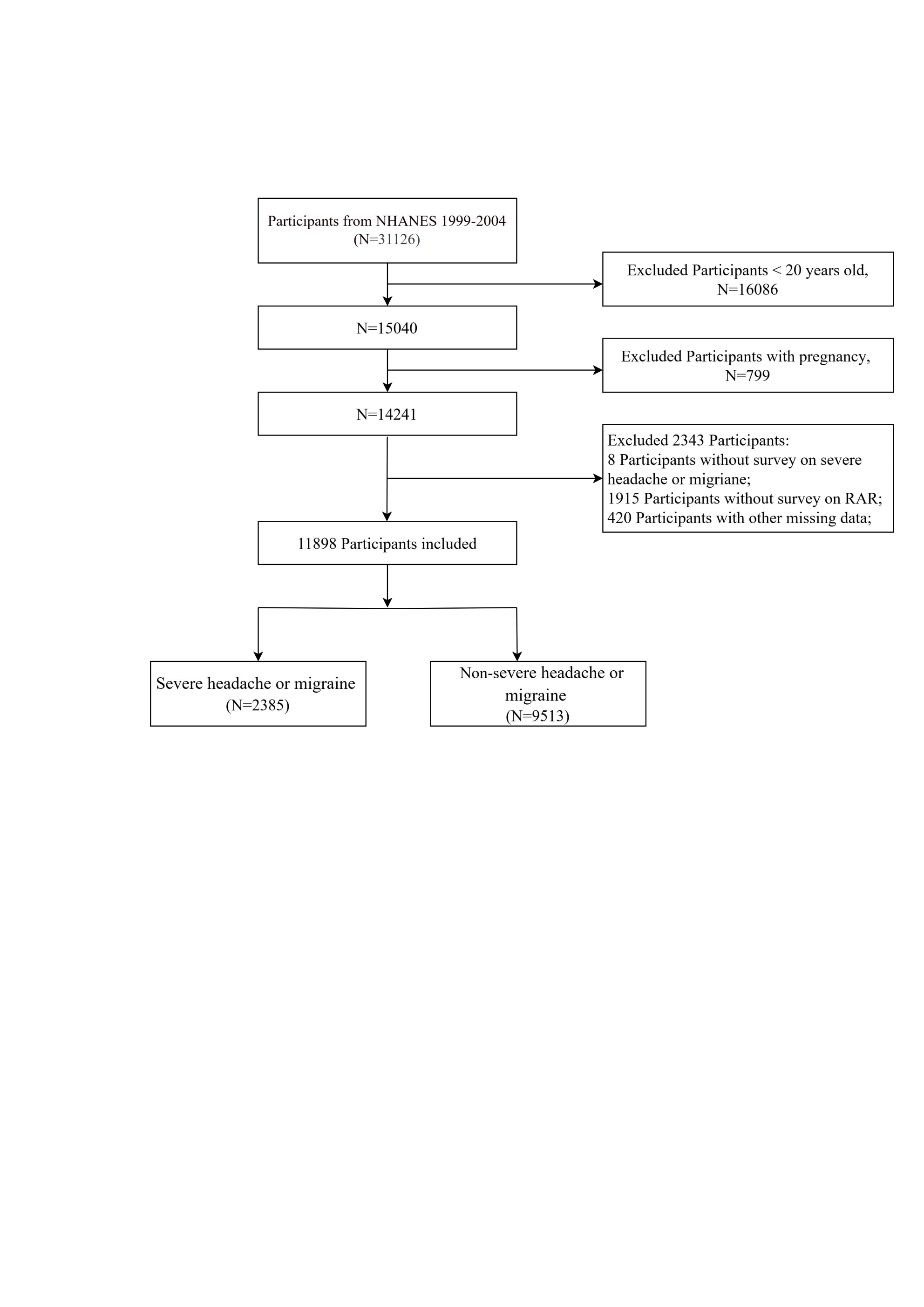

Supplement: Supplementary file 1 [file Image_1.tif]
