# Supplementary material for: The association between red blood cell distribution width to albumin ratio and migraine: evidence from clinical and population-based cohorts
Source: Front Neurol. 2026 May 12;17:1814482. doi: 10.3389/fneur.2026.1814482 (PMC13201144; doi:10.3389/fneur.2026.1814482)
Supplement: Supplementary file 2 [file Table_1.docx]

| **Supplementary Table 1** Basic characteristics of included participants (n = 11898) by the presence or absence of severe headache or migraine in the NHANES 1999-2004 | | | | |
| --- | --- | --- | --- | --- |
| Characteristic | Total (n=11898) | Severe headache or migraine(n=2385) | Non-severe headache or migraine (n=9513) | P value |
| Age(years) | 46.8 ± 16.4 | 42.6 ± 13.7 | 47.9 ± 16.9 | <0.001 |
| Gender (%) |  |  |  | <0.001 |
| Male | 5995 (50.39) | 829 (34.76) | 5166 (54.30) |  |
| Female | 5903 (49.61) | 1556 (65.24) | 4347 (45.70) |  |
| Race/Ethnicity (n,%) |  |  |  | <0.001 |
| Non-Hispanic White | 6111 (51.36) | 1090 (45.70) | 5021 (52.78) |  |
| Non-Hispanic Black | 2240 (18.83) | 498 (20.88) | 1742 (18.31) |  |
| Mexican American | 2689 (22.60) | 594 (24.91) | 2095 (22.02) |  |
| Other Hispanic | 529 (4.45) | 136 (5.70) | 393 (4.13) |  |
| Other Race | 329 (2.77) | 67 (2.81) | 262 (2.75) |  |
| Education status (n,%) |  |  |  | <0.001 |
| Less than high school | 3839 (32.27) | 852 (35.72) | 2987 (31.40) |  |
| High school diploma | 2813 (23.64) | 587 (24.61) | 2226 (23.40) |  |
| More than high school | 5227 (43.93) | 943 (39.54) | 4284 (45.03) |  |
| Not recorded | 19 (0.16) | 3 (0.13) | 16 (0.17) |  |
| Marital status (n,%) |  |  |  | <0.001 |
| Married | 6596 (55.44) | 1210 (50.73) | 5386 (56.62) |  |
| Widowed | 1160 (9.75) | 163 (6.83) | 997 (10.48) |  |
| Divorced | 1067 (8.97) | 243 (10.19) | 824 (8.66) |  |
| Separated | 374 (3.14) | 116 (4.86) | 258 (2.71) |  |
| Never married | 1684 (14.15) | 391 (16.39) | 1293 (13.59) |  |
| Living with partner | 618 (5.19) | 184 (7.71) | 434 (4.56) |  |
| Not recorded | 399 (3.35) | 78 (3.27) | 321 (3.37) |  |
| Poverty-income ratio (n,%) |  |  |  | <0.001 |
| ≤1.3 | 2991 (25.14) | 755 (31.66) | 2236 (23.50) |  |
| 1.3-3.5 | 4273 (35.91) | 847 (35.51) | 3426 (36.01) |  |
| > 3.5 | 3635 (30.55) | 578 (24.23) | 3057 (32.13) |  |
| Not recorded | 999 (8.40) | 205 (8.60) | 794 (8.35) |  |
| BMI(Kg/m^2^) | 28.1 ± 6.3 | 28.9 ± 7.0 | 27.9 ± 6.0 | <0.001 |
| Alcohol intake (n,%) |  |  |  | <0.001 |
| No | 3486 (29.30) | 809 (33.92) | 2677 (28.14) |  |
| Yes | 7735 (65.01) | 1419 (59.50) | 6316 (66.39) |  |
| Not recorded | 677 (5.69) | 157 (6.58) | 520 (5.47) |  |
| smoking status (n,%) |  |  |  | <0.001 |
| Never | 5965 (50.13) | 1218 (51.07) | 4747 (49.90) |  |
| current | 2662 (22.37) | 655 (27.46) | 2007 (21.10) |  |
| former | 3258 (27.38) | 510 (21.38) | 2748 (28.89) |  |
| Not recorded | 13 (0.11) | 2 (0.08) | 11 (0.12) |  |
| Physical activity (n,%) |  |  |  | <0.001 |
| Inactive | 5280 (44.38) | 1143 (47.92) | 4137 (43.49) |  |
| Moderate | 3284 (27.60) | 628 (26.33) | 2656 (27.92) |  |
| Vigorous | 3327 (27.96) | 612 (25.66) | 2715 (28.54) |  |
| Not recorded | 7 (0.06) | 2 (0.08) | 5 (0.05) |  |
| Hypertension (n,%) |  |  |  | 0.689 |
| No | 7909 (66.47) | 1598 (67.00) | 6311 (66.34) |  |
| Yes | 3872 (32.54) | 768 (32.20) | 3104 (32.63) |  |
| Not recorded | 117 (0.98) | 19 (0.80) | 98 (1.03) |  |
| Diabetes (n,%) |  |  |  | 0.086 |
| No | 10331 (86.83) | 2094 (87.80) | 8237 (86.59) |  |
| Yes | 1448 (12.17) | 263 (11.03) | 1185 (12.46) |  |
| Not recorded | 119 (1.00) | 28 (1.17) | 91 (0.96) |  |
| Coronary heart disease (n,%) |  |  |  | 0.035 |
| No | 11277 (94.78) | 2290 (96.02) | 8987 (94.47) |  |
| Yes | 551 (4.63) | 86 (3.61) | 465 (4.89) |  |
| Not recorded | 70 (0.59) | 9 (0.38) | 61 (0.64) |  |
| Stroke (n,%) |  |  |  | 0.006 |
| No | 11483 (96.51) | 2282 (95.68) | 9201 (96.72) |  |
| Yes | 402 (3.38) | 98 (4.11%) | 304 (3.20%) |  |
| Not recorded | 13 (0.11) | 5 (0.21%) | 8 (0.08%) |  |
| Laboratory measurements |  |  |  |  |
| WBC (10^9^/L) | 7.2 ± 2.2 | 7.2 ± 2.2 | 7.5 ± 2.2 | <0.001 |
| PLT (10^9^/L) | 267.7 ± 67.0 | 264.2 ± 64.9 | 280.4 ± 72.7 | <0.001 |
| LYM (10^9^/L) | 2.1 ± 1.1 | 2.1 ± 1.2 | 2.2 ± 0.9 | <0.001 |
| NEUT (10^9^/L) | 4.3 ± 1.6 | 4.3 ± 1.6 | 4.4 ± 1.7 | <0.001 |
| RDW (%) | 12.78 ± 1.17 | 12.69 ± 1.18 | 12.60 ± 1.03 | <0.001 |
| TC (mmol/L) | 11.27 ± 2.27 | 11.17 ± 2.25 | 11.29 ± 2.26 | 0.016 |
| TG (mmol/L) | 8.24 ± 6.12 | 8.07 ± 5.90 | 8.21 ± 6.59 | 0.503 |
| HDL (mmol/L) | 2.89 ± 0.87 | 2.86 ± 0.85 | 2.90 ± 0.88 | 0.022 |
| LDL (mmol/L) | 6.77 ± 1.96 | 6.63 ± 1.92 | 6.80 ± 1.96 | 0.009 |
| Albumin (g/dL） | 4.31 ± 0.33 | 4.30 ± 0.32 | 4.36 ± 0.33 | <0.001 |
| RAR | 2.9 ± 0.4 | 3.0 ± 0.42.9 ± 0.4 | 2.9 ± 0.43.0 ± 0.4 | <0.001 |
| **Note:** Values are weighted mean ± standard error (SE) or weighted frequency (%). P values are weighted. **Abbreviation:** BMI, body mass index; NHANES, National Health and Nutrition Examination Survey; RAR, ratio of red blood cell distribution width to albumin; RDW, red cell distribution width; HDL, high density lipoprotein; LDL, low density lipoprotein; LYM, lymphocyte; NEUT, neutrophil; PLT, Platelets; WBC, white blood cell; TC, total cholesterol; TG, triglyceride. | | | | |
